# Barriers and facilitators to implementing community-based physical activity interventions: a qualitative systematic review

**DOI:** 10.1186/s12966-021-01177-w

**Published:** 2021-09-07

**Authors:** Jemima Cooper, Joey Murphy, Catherine Woods, Femke Van Nassau, Aisling McGrath, David Callaghan, Paula Carroll, Paul Kelly, Niamh Murphy, Marie Murphy, Irish Physical Activity Research Collaboration (I-PARC), Adrian Bauman, Benny Cullen, Colette Brolly, Enrique García Bengoechea, Fiona Mansergh, Grainne O’Donoghue, James Lavelle, Nanette Mutrie, Niamh Barry, Peter Smyth, Ronan Kielt, Sarah O’Brien, Shirley O’Shea, Vydehi Muppavarapu

**Affiliations:** 1 Physical Activity for Health Research Cluster, Physical Education & Sport Sciences Department, University of Limerick, Limerick, Ireland; 2 Health Research Institute, University of Limerick, Limerick, Ireland; 3 Amsterdam UMC, Vrije Universiteit Amsterdam, Department of Public and Occupational Health, Amsterdam Public Health research institute, Amsterdam, The Netherlands; 4 Department of Sport and Exercise Science, Waterford Institute of Technology, Waterford, Ireland; 5 Sport Ireland, Dublin, Ireland; 6 Physical Activity for Health Research Centre, University of Edinburgh, Edinburgh, UK; 7 Centre for Exercise Medicine, Physical Activity and Health, Ulster University, Belfast, Northern Ireland

**Keywords:** Implementation factors, Physical health, Real-world, CFIR, Systematic review, Physical activity, Community, Intervention

## Abstract

**Background:**

Over the past decade several physical activity (PA) interventions have been shown to be efficacious in a controlled research setting, however there is a continued lack of evidence for how to successfully implement these PA interventions in real-world settings such as the community. This review aims to explore the barriers and facilitators that affect the implementation of community-based PA interventions and make recommendations to improve implementation from the included studies.

**Methods:**

A systematic literature search of EBSCOhost, Scopus, PUBMED and Web of Science was conducted to identify articles that reported qualitative data on the implementation factors of community-based interventions where PA was a primary outcome. Data were extracted using the Consolidated Framework for Implementation Research (CFIR) as a guide. Implementation factors and recommendations were then mapped onto the 5 domains of the CFIR and synthesised thematically.

**Results:**

From 495 articles, a total of 13 eligible studies were identified, with 6 studies using a mixed methods approach, and 7 reporting qualitative methods only. There were 82 implementation factors identified, including 37 barriers and 45 facilitators, and a further 26 recommendations from the papers across all 5 domains of the CFIR. More barriers than facilitators were identified within the CFIR domain inner setting, in contrast to all other domains where facilitator numbers outweighed barriers.

**Conclusions:**

This review identified many facilitators and barriers of implementing physical activity interventions in the community. A key finding of this review was the impact of implementation strategies on successful implementation of community PA interventions. From the evidence, it was clear that many barriers to implementation could have been negated or reduced by an implementation plan in which several strategies are embedded. The findings of this review also suggest more attention to individual’ skills and involvement is needed to improve self-efficacy and knowledge. The role of individuals across all organisational levels, from providers to leaders, can impact on the implementation of an intervention and its success.

**Trial registration:**

PROSPERO - CRD42020153821.

**Supplementary Information:**

The online version contains supplementary material available at 10.1186/s12966-021-01177-w.

## Introduction

Insufficient physical activity (PA), defined as not engaging in at least 150 min of moderate-intensity PA or 75 min of vigorous-intensity PA per week [1], is widely acknowledged as a global pandemic [1, 2], prevalent in almost a third of adults worldwide [3], and responsible for 6.4% of global premature mortality [4]. Engaging in sufficient levels of PA is associated with a host of physical and mental health benefits, such as a reduced risk of non-communicable diseases like cancer, Type 2 Diabetes and cardiovascular disease [2], as well as alleviating the impact of mental health disorders such as depression and anxiety [5]. In addition, PA has been found to have benefits to social health, such as contributing to community cohesion [6]. As a result, increasing global PA levels would have a substantial positive impact on population health.

In the past decade, there has been a marked increase in the number of efficacious PA interventions aiming to address this global issue of insufficient PA [7]. Despite this, there is a continued lack of evidence for successful interventions in real-world settings, such as the community, as only a small number of interventions move from research into practice [8]. The term ‘community-based’ has a wide range of meanings in the literature, however McLeroy et al. [9] define community-based interventions as comprising of 4 categories: community as a setting (i.e. geographically defined area), community as a target (i.e. engaging community members), community as an agent (i.e. respecting and using existing natural capacities of the community), and community as a resource (i.e. use of community resources such as ownership to enact change). McLeroy et al. acknowledge the difficulty of summarising results within these categories, and that many community interventions have characteristics from multiple categories [9]. Key elements of community-based interventions emphasise social interactions [10], include the mobilisation of communities to actively participate in achieving the intervention goals and include implementing activities in community settings such as workplaces, places of worship, health care facilities, and schools [11]. Community engagement is recognised as a critical component of public health strategies where communities can take control and be owners of their own destinies [12]. Furthermore, the International Society for Physical Activity and Health (ISPAH) has recently published the ‘Eight Investments That Work For Physical Activity’ 2020 [13], with the implementation of community-wide programmes identified as one of the eight key investments, further highlighting how important they are for supporting the global target of a reduction in insufficient PA.

Implementation can be defined as the process of integrating an intervention into practice within a specific setting [14]. Implementation science is an emerging area within PA research aimed at promoting the systematic uptake of evidence-based practice into routine practice to improve quality and effectiveness [15, 16]. However, of the studies published within this area, only 20% examined the implementation of effective PA interventions in real-world settings [17], highlighting a significant gap between research and practice. This research-to-practice gap, whereby there is a lack of transfer and translation of interventions successful in controlled conditions into real-world contexts [17], poses a significant challenge for public health and community-engaged researchers and practitioners attempting to implement and scale-up effective PA interventions for population health [18]. Furthermore, a majority of the limited real-world implementation research within PA comes from clinical and healthcare settings, and it is unclear if these findings can be translated where the delivery context includes a wide variety of community settings [8]. As such, there is a research-to-practice evidence gap within the implementation of effective PA interventions in real-world settings, which needs to be addressed given the implications for improving public health if successful PA interventions are implemented [8, 19].

In order to address this gap, current literature highlights the need to develop and communicate implementation strategies for PA interventions across sectors and disciplines [19, 20]. However, in order to develop such strategies, Naylor et al. [15] suggest that we must first understand the factors related to implementing PA interventions, which is the cornerstone to successfully integrating PA interventions into community settings. The implementation of PA interventions in real-world settings, such as the community, is a complex and challenging process, with multilevel factors influencing effective implementation [8, 14]. The Consolidated Framework for Implementation Research (CFIR) [21] is a commonly used determinant framework in implementation research [22]. It has been described as a ‘meta-theory’ as it synthesises multiple implementation theories into accessible domains and constructs that are practical and easily applicable to the area of PA intervention research. The framework comprises of 39 constructs across five domains including 1) intervention characteristics, 2) inner setting, 3) outer setting, 4) individual characteristics and 5) processes of implementation [21]. Due to the broad nature of this framework, the CFIR could be useful to aid the understanding and synthesis of implementation factors identified in the literature.

Few reviews report implementation factors of PA interventions in practice [15, 23, 24], defined as taking place in the real world, but none focused specifically on PA interventions across community-based settings. Instead previous reviews have focused solely on the school setting with Naylor et al. [15] recommending the use of comprehensive framework to help identify any domains of implementation that may be overlooked in the current literature. Furthermore, within this limited number of existing reviews of implementation factors of PA interventions in practice, none were qualitative reviews; including either mixed methods or solely quantitative data. Literature highlights that qualitative research is needed to increase knowledge of health intervention assumptions [25], components [26], and the active mechanisms which influence implementation. Furthermore, a qualitative approach allows for in-depth insight into individual’s perceptions of the barriers and facilitators that are relevant to their context and is increasingly recognised as an important approach for developing the evidence base in PA and implementation research [27, 28]. Thirsk et al. [29] commented on this stating that qualitative methods to research complex health interventions are underdeveloped. Therefore, the aim of this study was to review the available qualitative research reporting on facilitators and barriers of implementation for interventions which promote PA in community-based settings. A secondary aim of this work was to provide recommendations to improve implementation identified from the included studies.

## Methods

This qualitative systematic review was undertaken as part of the work of the Irish Physical Activity Research Collaboration (I-PARC); a multisectoral collaboration established in 2018 to foster insight, intelligence and innovation to enable more people in Ireland to be more active, more often [30]. The review was undertaken according to the Preferred Reporting Items for Systematic Reviews and Meta-Analyses (PRISMA) checklist [31] and was prospectively registered with PROSPERO, under registration number: CRD42020153821.

### Literature search

A systematic literature search was undertaken of databases including EBSCOhost (Academic Search Complete, MEDLINE, PsycINFO, SPORTDiscus with Full text, UK & Ireland Reference Centre), Scopus, PUBMED and Web of Science in February 2020. The search aimed to find articles with information relating to our study aim. The databases were searched for articles that included terms related to the main concepts: ‘implementation’, ‘barriers’, ‘facilitators’, ‘physical activity’, ‘community’, and ‘interventions’. Relevant MeSH, non-MeSH and Thesaurus terms were used, and Boolean operators were used to link the keywords. The search syntax was adapted to suit each database. Non-intervention studies such as reviews, protocol papers, commentaries and editorials were excluded, as were full-text articles not in English. The year 2000 was chosen as the cut-off for this review, as the authors found that the majority of relevant articles have been published after this year. A PubMed generated histogram of ‘Results by year’ for ‘physical activity interventions’ confirms this, showing that the majority of research in this field is published from 2000 onwards, with the number of articles increasing greatly from then. Articles including interventions targeting active travel and rehabilitation were excluded through the search strategy by excluding articles including terms related to the concepts of ‘active travel’ and ‘rehabilitation’, as they do not fit under the definition of a community-based intervention used for this review as per McLeroy et al. [9]. A librarian and the full author team were consulted to develop the final search strategy, which can be found in Additional file 1, and details the search syntax groups and syntax combinations.

Titles and abstracts of studies identified through the search strategy were imported into Endnote, and duplicates were removed. The remaining studies were then imported into Rayyan QCRI, where they were independently evaluated for eligibility based on four key inclusion criteria by first reviewer (JC) and second reviewer (JM). The inclusion criteria included studies which 1) used a qualitative study design, 2) reported on an intervention delivered in a community-based setting, 3) included PA promotion as a primary outcome, and 4) reported on factors related to intervention implementation. Studies specifically targeting populations with a disability or mental health challenges were excluded from this review as the authors believed they present their own unique barriers and facilitators. Both reviewed half the studies, then assessed 25% of each-other’s studies to ensure agreement. The reviewers had an agreement rate of 97%, with a third reviewer (CW) consulted where disagreements arose regarding eligibility of articles. At the full-text review stage, the remaining articles were independently screened for eligibility by first reviewer (JC) and second reviewer (JM) using the same technique as the previous stage, leading to an agreement rate of 91%, with a third reviewer (CW) consulted where conflicts on article inclusion remained.

### Data extraction

Articles that met all four inclusion criteria were compiled and data were extracted into a standardised data extraction excel spreadsheet (see Additional file 2). This detailed study characteristics (author/year, country, main study aims and outcomes, study participants, setting, sample size, intervention aims, intervention outcome, study design, qualitative data collection measures, implementers, and frameworks used to report) in addition to the barriers, facilitators and recommendations extracted under the five domains of the CFIR [21] which are highlighted in the introduction. To synthesise results across studies, standardised terminology was adopted. ‘Providers’ are those responsible for the delivery of the intervention often working face-to-face with participants, ‘leaders’ are those in management or leadership positions responsible for coordination of the intervention or in senior positions within the implementing organisation, ‘staff’ refers to all staff within an organisation across the different levels of hierarchy, and ‘stakeholders’ refers to any individuals or partner organisations with a role in the intervention.

First reviewer (JC) did the initial extraction, and reviewers JM, CW and FvN were consulted to gain consensus on the deductive theming of data under each CFIR domain, guided by the constructs of CFIR. Further feedback was given on the theming of data from the full author team during the paper conceptualisation and drafting stages. A risk of bias assessment was conducted by first reviewer JC, and further assessed by second reviewer JM, using the Mixed Methods Assessment Tool (MMAT) [32]. MMAT is a critical appraisal tool for assessing the quality of studies including two screening questions and five questions on core methodological criteria with response options of “yes”, “no” or “can’t tell”. Hong et al. [32] discourage the calculation of an overall score from the core criteria and exclusion of any low-quality studies, but rather suggest providing a more detailed assessment and understanding of each study’s quality, which was conducted with the studies in this review.

### Data analysis

A qualitative reflexive thematic analysis was conducted whereby the barriers, facilitators and recommendations within each CFIR domain were inductively synthesised into sub-themes. A key feature of thematic analysis is the development of descriptive and analytical themes that translate findings across studies, and was first applied to a review of barriers and facilitators, indicating its suitable use as an analytical approach in this review [33]. The thematic analysis was undertaken according to the 6 phases of reflexive thematic analysis outlined by Braun and Clarke [34]: 1) familiarisation; 2) data coding; 3) generating initial themes; 4) reviewing & developing themes; 5) refining, defining and naming themes; 6) writing the report. First reviewer JC followed these six steps, starting with familiarisation of the data, which occurred throughout screening, data extraction and analysis stages, through multiple readings of the included studies. First reviewer JC conferred with reviewers JM and CW during the data coding stage 2 to ensure consensus on coding practices. JC generated initial themes from the data in step 3, then conferred again with authors JM and CW when reviewing and developing themes in step 4. Then when themes were refined, defined and named during step 5, reviewer FvN, who has extensive implementation experience, was consulted for feedback and consensus on the naming and grouping of sub-themes under each of the CFIR domains. During the sixth step, writing the report, all authors gave feedback on the placement of the sub-themes under each of the CFIR domains, and the naming of the themes, and whether the themes accurately represented the supporting data. This was to ensure author consensus, and credibility and trustworthiness of the findings. As Braun and Clarke [35] highlight, in qualitative research, each researcher brings their own unique insight and experience to the process, thus in ensuring all authors were involved during the analysis stage, this allowed for a more rich and well-rounded analysis of the data.

## Results

**Fig. 1:**
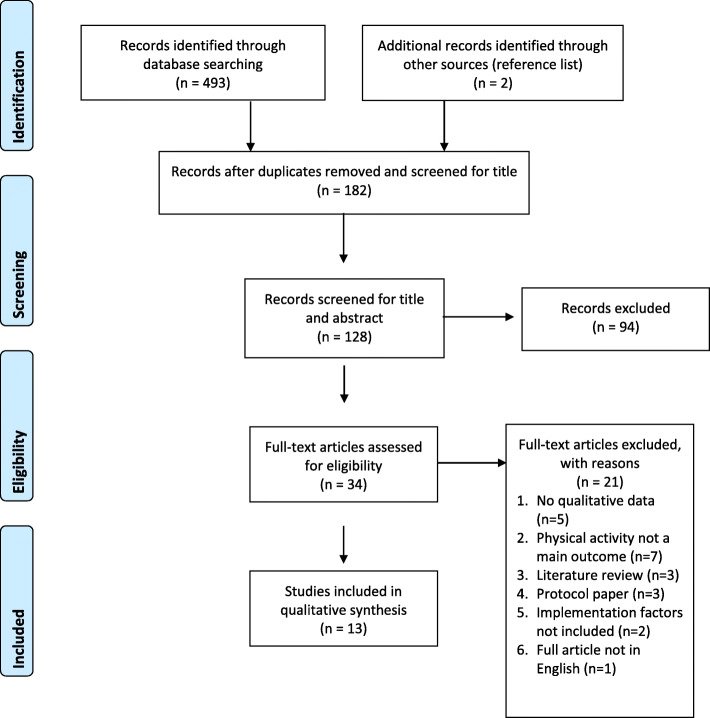
PRISMA

The search strategy (Additional file 1) resulted in a total of 495 articles identified. After duplicates were removed and articles were screened for title and abstract, 34 full-text articles were deemed eligible for full-text screening. During full-text screening, a total of 21 articles were excluded as they did not meet the inclusion criteria. Thirteen studies were included in the qualitative extraction and synthesis. Figure 1 shows a PRISMA flow diagram of this process.

From the 13 studies included, 6 studies used a mixed methods approach, and 7 reported qualitative methods only. The 13 studies reported interventions based in eight different countries: Canada (*n* = 2), Denmark (*n* = 1), France (*n* = 2), Spain (*n* = 2), South Africa (*n* = 1), The Netherlands (*n* = 1), United Kingdom (*n* = 3) and United States of America (*n* = 1). There were 11 different interventions covered by these 13 studies. These included the implementation of a church-based intervention [36], school-based communities [37–41], communities targeting populations of specific demographics [19, 42–44] and community healthcare interventions [45–47]. When classifying studies by type of PA intervention, the majority included PA as a main outcome in conjunction with other health enhancing outcomes, such as healthy eating and smoking cessation. The lead agency implementing these PA interventions, often in partnership with other organisations, ranged from regional health agencies, to church leaders, regional education authorities, municipal policy and city district sports coordinators, county councils, walking promotion agencies and government ministries. For a more detailed overview of the characteristics of the studies included in this review, see Additional file 2.

Risk of bias has been reported in Additional file 2. All studies scored “yes” for the two screening questions, indicating that the MMAT was suitable to assess them. All studies also scored “yes” on the 5 methodological criteria. Additionally, as encouraged by Hong et al. [32], a detailed assessment of the studies was undertaken in addition to the scoring, with observations of why the studies scored yes also recorded.

### Implementation factors

Table 1 provides a summary of the implementation factors grouped by CFIR domain. A more detailed report of the factors and supporting data is included in Additional file 3. We identified 82 implementation factors, consisting of 37 barriers and 45 facilitators, which are reported as per the 5 domains of the CFIR.

**Table 1:** Facilitators & Barriers of Implementation

Barriers	Facilitators
1- **Intervention Characteristics**	
B1.1 Name of intervention B1.2 Lack of evidence base B1.3 Adaptability – conflict between standardise vs tailoring to context B1.4 Lack of resources B1.5 Safety consideration B1.6 Physical and temporal barriers B1.7 Failures in new technology implemented	F1.1 Cost to participant F1.2 Cost to organisation F1.3 Pragmatic and clear programme content F1.4 Adaptability (both by implementers and to context) F1.5 Programme compatibility with staff and participants F1.6 Development and availability of innovative information and communication technologies F1.7 Credibility from evidence source F1.8 Positive perception of intervention implementer by participants F1.9 Sustainability of the intervention
2- **Inner Setting**	
B2.1 Competing priorities B2.2 High staff turnover B2.3 Lack of communication within the team B2.4 Lack of support from leadership B2.5 Lack of funding B2.6 Implementing intervention from obligation B2.7 Staff burnout B2.8 Lack of perceived responsibility and motivation among organisations B2.9 Limited capacity to take part in multiple initiatives B2.10 High level of organisation and administration needed B2.11 Too much change required to implement B2.12 Poor staff training quality	F2.1 Strong commitment from leadership F2.2 Clear information and communication strategies within organisations F2.3 Provider training and capacity building F2.4 Strong shared commitment and sense of ownership F2.5 Feedback to staff F2.6 Easy to integrate intervention goals within existing structures F2.7 Strong staff relationships
3- **Outer Setting**	
B3.1 Cultural barriers B3.2 Instability or lack of policies supporting target group B3.3 Poor relationship between organisation and community B3.4 Lack of community buy-in B3.5 Lack of coordination and communication between organisations B3.6 Funding between collaborating organisations B3.7 Availability of resources	F3.1 Participation of stakeholders in decision-making process F3.2 Funding F3.3 Accessible to communities in which intervention was implemented F3.4 Community involvement to support the intervention F3.5 High perceived fit of intervention in policy goals/agendas F3.6 Political advocacy and support F3.7 Effective communication strategies between stakeholders F3.8 Volunteerism F3.9 Role of support and research system F3.10 Leadership and buy-in from range of stakeholders
4- **Individual Characteristics**	
B4.1 Lack of motivation B4.2 Lack of knowledge B4.3 Perceived imposed participation in intervention training B4.4 Values inconsistent with lifestyle/context B4.5 Lack of perceived importance of communicating with participants by organisation staff B4.6 Perceived workload among staff B4.7 Poor participant attitude B4.8 Challenge to find committed leaders	F4.1 Well-trained F4.2 Dedicated F4.3 Leaders take ownership of problem addressed by intervention F4.4 Leaders motivate others F4.5 High individual motivation of staff F4.6 High perceived importance of intervention by staff F4.7 Positive attitudes and beliefs F4.8 Members and leaders experienced increases in self-efficacy F4.9 Feeling empowered F4.10 Strong feeling of reward from engaging across all levels
5- **Processes of Implementation**	
B5.1 Insufficient resources allocated for implementation B5.2 Complexity of intervention B5.3 Top-down implementation strategy	F5.1 Engaging key stakeholders in decision-making throughout whole implementation process (Including pre-delivery of intervention) F5.2 Appointed community-based members as leaders F5.3 Designed using existing resources and context characteristics F5.4 Involvement of experts to tailor intervention F5.5 Using theoretical model to inform recruitment strategies F5.6 Support and research staff checking in with program staff facilitated problem solving and feedback loops F5.7 Use of wide variety of strategies to implement the intervention F5.8 Enough time for preparation before delivery F5.9 Collaborative effort built into design

### Intervention characteristics

The degree to which an intervention can be adapted, tailored, refined, or reinvented to meet local needs was highlighted by seven studies [19, 36, 38, 39, 41, 43, 44] as a facilitator for implementation. For instance, providers were able to make minor changes to the intervention while retaining core components to facilitate implementation, such as editing content due to time constraints and context-specific health activities. In addition, the potential for tailoring the intervention allowed providers to refine recruitment strategies to suit the target population and context, and enhance the suitability of the content and delivery style for the target group. Programme compatibility was a facilitating intervention characteristic identified by five studies [19, 39, 40, 42, 43]. The compatibility of intervention tasks with provider’s regular function facilitated implementation, as did interventions that fit easily within participant’s lifestyles. Furthermore, the timeliness, relevance, geographical accessibility and uniqueness of the intervention within communities all contributed to the compatibility of the intervention for participants, which in turn facilitated implementation.

In addition to being a facilitator, adaptability was also mentioned by four studies as a barrier to implementation [38–40, 46], due to the conflict between standardising the intervention versus tailoring it to context. For instance, there is a discrepancy between the need to standardise and maintain complexity of the intervention in order to ensure fidelity and maintain intervention effect, and the need to tailor the implementation strategies to the local cultural, social and environmental context to ensure compatibility, as a uniform approach may not translate to all contexts. Additionally, adaptation results in a variety of implementation practices in non-trial settings, due to various contextual constraints, which can lead the intervention to require extensive input from a public health department to address, and may subsequently impact fidelity and quality [40]. Other barriers within the intervention characteristics domain included a lack of clarity in the name and the lack of evidence base for the intervention.

### Inner setting

A sense of ownership from staff was found to be fundamental to successful implementation, with strong commitment and motivation from the organisation to comply with a shared goal also identified as a contributing factor [37, 39, 43]. Furthermore, an existing culture of staff working together within the organisation helped to facilitate implementation, along with a sense of belonging within each branch of the organisation involved in implementing the intervention. In particular, support and commitment from leaders was a facilitating factor identified in three studies [36, 37, 43]. Buy-in from leaders was an essential factor for successful implementation, and in organisations where existing projects were extended, support from project leaders promoted sustainability. Leaders also acted as role models within their organisations, helping to facilitate implementation and taking the lead in promoting physical health. Studies (*n* = 2) also cited being able to easily integrate intervention goals, methods, procedures and tasks within existing structures as aiding implementation [19, 39].

Four studies reported competing priorities as an implementation barrier within the inner setting [36, 37, 40, 44]. For example, competing demands and expectations of organisations were reported as a major barrier to implementation that can shift the focus from PA and health interventions towards other priorities. In some cases, conflicts of interest also emerged among implementers of the intervention where agendas and goals did not align. Studies (*n* = 4) also highlighted the implementation of the intervention requiring a high level of organisation as an internal barrier [39, 41, 44, 46]. Complex interventions needing a high level of organisation cause confusion among implementers in instances where this level organisation is lacking. Such confusion manifests itself as a lack of effective delivery of training to staff, or an underestimation of the coordination and effort needed to attract participants, leading to insufficient time being allocated for preparation [39].

### Outer setting

Effective communication strategies between stakeholders was a key facilitator within the outer setting as identified in four studies [38, 39, 43, 44]. For instance, the formalisation of partnerships and continued engagement of all partners created a sense of unity. Effective communication between stakeholders facilitated trust and motivation between partner organisations and also participants. Studies (*n* = 5) also reported community involvement as a facilitator [36, 37, 40, 43, 45]. Community members’ motivation and commitment helped facilitate the delivery of the intervention and to link with community resources, in addition to support from local authorities. Alongside the role of community, political advocacy and support was also acknowledged as an important outer setting facilitator [19, 37–39]. Aligning the intervention with objectives of relevant government departments was reported to be essential for scale-up and sustainability. Furthermore, governmental and political support was found to promote active community participation in the intervention, and aided initiation of collaborations between policy makers, practitioners and researchers.

Poor availability and accessibility of the necessary facilities, including affordability, and a lack of resources, all acted as a key barrier within the outer setting [39, 43, 44]. Both the lack of community buy-in and a poor relationship between the implementing organisation and the community also acted as common barriers [38, 39, 41, 43, 45]. For instance, failures to communicate the importance of the intervention outcomes to the participants within the community was denoted by a lack of community buy-in. Furthermore, a weak relationship between the implementing organisation and the community could not be strengthened even when the intervention was initiated by a community-made decision.

### Individual characteristics

Suitably trained individuals involved in implementation were a facilitating factor to implementation in five studies [38, 39, 41–43]. The inclusion of training led to a positive effect on staff, who felt that the intervention was in line with their expectations, and who then had realistic expectations about tasks and responsibilities. Additionally, being well-trained was significant for individual motivation to implement and maintain an intervention, and meant that individuals understood the core principles of the intervention. Positive attitudes and beliefs were another key facilitator of implementation in the individual characteristics domain [19, 39, 43]. This was found to be important across different levels of stakeholders, from implementation staff to political stakeholders. In particular, positive attitudes and encouragement from providers had a big impact on participant engagement however more broadly, it facilitated adaptations and changes in policies and practices throughout implementation. A further facilitator was a sense of reward from engaging in the intervention across all levels of stakeholders, from participants, to providers, leaders and partner organisations [19, 36, 40, 43]. Stakeholders experienced a range of perceived benefits from engaging with the intervention, which resulted in buy-in and a sense of ownership.

Studies reported that where individuals involved in the implementation of the intervention; lacked knowledge [37], placed low sense of value on the intervention [38] or perceived involvement as a high workload, were barriers to success [39]. Additionally, poor participant attitude can also act as a barrier for successful implementation [39].

### Processes of implementation

Five studies described a key facilitating factor of the implementation process as engaging key stakeholders in decision-making throughout the whole implementation process, including pre-delivery of the intervention [19, 36, 43, 45, 46]. For instance, co-designing the intervention with community partners prior to implementation meant that communities were empowered through a consultation process that identified their needs. Furthermore, using a bottom-up process of involving key stakeholders in the decision-making process about priorities according to their specific context, and the roles and contributions of members, can lead to greater intervention commitment and adherence, and an enhanced sense of ownership. Other facilitators for effective implementation included designing the intervention to suit the characteristics of the context and to utilise existing resources [37, 43]. In addition, the use of existing community infrastructure was seen to facilitate the intervention implementation process.

There were three factors that acted as barriers to the implementation process: insufficient resources allocated for implementation, complexity of evaluation, and a top-down implementation strategy. For instance, the complexity of tracking the intervention for evaluation purposes was beyond the capacity of the providers and impacted adoption [46]. Where a bottom-up approach acted as a facilitator, a top-down implementation strategy was a barrier, with top-down policies perceived negatively at local-level and conflicting with local needs and contexts [38].

**Table 2:** Recommendations for Implementation

1- **Intervention Characteristics**	
R1.1 Policy can be a formidable tool for health promotion R1.2 Potential barriers need to be anticipated and addressed before implementation R1.3 Programme components - standardised, simple, scalable and renewed R1.4 Maintain cost to participant (do not increase) R1.5 Plan for scale up R1.6 Innovative ways of motivating participants not ready to change and ensuring continuity of care for those with intention to change behaviour to minimize false expectations R1.7 Using ongoing research to assist organisations in maintaining fidelity to core principles R1.8 Assess fidelity of delivery	
2- **Inner Setting**	
R2.1 Improvement of efficiency and reliability of the information and communication tools and databases	
3- **Outer Setting**	
R3.1 Need for local support R3.2 Need for policy development R3.3 Needs long-term funding R3.4 Formalised coalition R3.5 Increase media support R3.6 Easier communication to participants R3.7 Improve coordination to avoid duplication R3.8 Need to work in partnership with organisations and agencies working in target groups, especially in hard to recruit groups R3.9 More research focus is needed on fidelity to implementation strategies	
4- **Individual Characteristics**	
R4.1 More attention for stakeholders’ skills and involvement across contexts is recommended to improve self-efficacy	
5- **Processes of Implementation**	
R5.1 Planning with clear steps for implementation R5.2 Collaboration between all aspects of community and setting from start of programme implementation and before programme is introduced. This introduces complexity to the process R5.3 Use of social marketing principles R5.4 Maintain program champion R5.5 Maintain ease of delivery R5.6 Understanding if and how implementation decisions are made and what trade-offs are made at the different levels of the intervention is important for understanding intervention implementation R5.7 Intensity of contact between research team and providers may have contributed to level of adherence	

### Recommendations

In addition to extracting the barriers and facilitators for each CFIR domain, recommendations for implementation from the studies were also extracted. Table 2 presents this data, which includes 26 recommendations from 9 of the 13 included papers. No paper alone provided recommendations across all 5 domains. Full recommendations with supporting data can be found in Additional file 4.

Recommendations from the outer setting (*n* = 9) and intervention characteristics (*N* = 8) were the most identified. In the outer setting, studies recommended the need to improve coordination and communication at community level to avoid duplication and improving effective use of resources [45]. Other studies mentioned the need for local support and consultancy services and a need for more research focused on fidelity to implementation strategies [37, 42]. Within the intervention characteristics, policy was recommended as a potentially formidable tool for health promotion, if policies are developed with an understanding of implementation in the local context [37]. Another study recommended that potential barriers need to be anticipated and addressed prior to implementation, through an extensive assessment of the intervention context [38]. Two studies recommended that the programme components are standardised, simple, scalable and regularly renewed so the intervention doesn’t seem repetitive [39, 42].

## Discussion

The primary aim of this study was to explore the existing qualitative research concerning the barriers and facilitators to implementing community-based PA interventions as well as recommendations to improve implementation. The studies included provided evidence of the barriers and facilitators of implementing PA interventions in the community. In this review, the CFIR was used to identify and synthesise implementation factors and recommendations across its five domains, resulting in a useful and structured overview of the current qualitative literature for future researchers to supplement existing quantitative research, and inform the implementation of community-based PA interventions. There was a relatively even distribution of factors across the domains, and between barriers and facilitators, demonstrating that the studies included in this review explored a wide range of the different aspects of implementing PA interventions.

A secondary aim of this study was to identify recommendations from the existing research, of which 26 were found. Many recommendations were practical, and highlighted key areas within the 5 CFIR domains to target to improve implementation. In particular, recommendations for the outer setting and processes of implementation supported the need for clear implementation strategies and coordination across stakeholders, and offered suggestions such as negotiated planning and stepwise implementation.

A comprehensive understanding of barriers and facilitators of PA interventions should provide an evidence-base from which to develop effective implementation strategies, however there is an absence of an international synthesis of this literature [23]. For example, one interesting finding was that there were more barriers than facilitators identified within the CFIR domain inner setting. This contrasts with all other CFIR domains, and indicates that during the implementation process, the inner setting is of key importance. Any organisation implementing community-based PA interventions need to take time to anticipate and address these barriers from the start. Furthermore, several barriers have multiple consequences which impact on, and exacerbate, other barriers. For instance, staff burnout is a barrier to implementation which may also contribute to high staff turnover (also identified as a barrier). Attempting to address barriers in the inner setting can reduce this potential negative domino effect. Given the lack of qualitative implementation literature of community-based interventions, it is difficult to conclude that this finding is shared by other related studies. However, there is evidence relating more generally to PA implementation, that inner setting factors, such as a lack of resources and appropriately trained staff members, can greatly affect how interventions are implemented [48].

The impact of implementation strategies was another key finding of this review. From the evidence, it was clear that many barriers to implementation could have been negated or reduced by an implementation plan in which several evidence-based strategies are embedded [49]. Interventions will not implement themselves, and as such, evidence-based strategies are needed for each stage of the process from preparation through to delivery and evaluation. This finding is supported by Morrison et al. [50] who reported that a clear, succinct summary of the intervention plan both enhances and facilitates communication among stakeholders. Furthermore, Powell and colleagues [51] found that implementation strategies often lack the level of detail needed for effective use in real-world settings, and even selecting appropriate strategies is a complex task. A recommendation from this review is the need to give careful consideration to the way interventions are delivered, and the importance of negotiated planning [38]. The wider literature has acknowledged that although the terms and definitions for implementation strategies were unclear in the past, leading to confusion when selecting appropriate strategies, further research has since provided more clarity. There are now several clear frameworks including Expert Recommendations for Implementing Change (ERIC) [49, 51, 52], and the Practical planning for Implementation and Scale-up (PRACTIS) guide specific to PA interventions, which provides practical guidance for researchers on how to effectively plan implementation and scale-up [8]. Thus, future studies have access to emerging implementation strategies and should utilise these to address the persistent barrier of a lack of an implementation plan. However, it must also be acknowledged that there is a lack of evidence of the effectiveness of existing implementation strategies, thus further research to test these strategies is needed.

Individual characteristics are an often-overlooked domain within implementation research, and as such, the impact of the role of individuals in the successful implementation of an intervention are undervalued. There is a lack of consideration when developing implementation strategies for the need to create buy-in and change from individuals at all levels of responsibility within an organisation. This is evidenced by findings from the studies included in this review, which state that organisations can provide training on practical delivery, but providers need to see the benefits and make changes to their own behaviours in order to successfully implement an intervention. When individuals implementing the intervention perceive a sense of reward and recognise the value of the intervention, these act as key facilitators. Inner and outer setting factors could be contributing to individual characteristics being undervalued, as barriers such as high staff turnover, poor staff training quality, lack of communication within teams, and poor relationship between community and organisation all contribute to the inability to invest in individual-level capacity building. Furthermore, the results highlight how facilitators in one domain support those in another, and at the core of that support is the individuals at the different levels within an organisation, from providers through to leadership level, and other key stakeholders such as participants, external funders and community leaders. In order to address this understudied factor, the findings of this review suggest more attention to providers’ skills and involvement is needed to improve self-efficacy and knowledge. This is supported by Powell et al. [51] who highlight the need to assess the factors that influence implementation processes prior to delivery, which includes the characteristics and preferences of individuals involved in both the delivery and receipt of the intervention. However, Byrne [53] accepts that while engaging key stakeholders such as providers and participants as part of the implementation strategy appears to be a promising approach, it is concluded that evidence is sparse, and more guidance is needed to effectively use this approach.

The data presented within this review highlights what is currently known in relation to the research-practice gap of implementation science within PA research [8, 23], while highlighting key priorities moving forward. The findings suggest that the barriers and facilitators within community-based settings may be transferable to other practice-based PA interventions, as the implementation factors found are similar to those identified by Cassar et al. [24] in their systematic review. This suggests that although the context of the intervention can differ, similar factors play a role in the implementation process. Furthermore, the broad definition of community used in the review [9] resulted in the qualitative data included within this review being synthesised and translated across a wide variety of community settings, further facilitating the transferability of the findings.

This systematic review found only 13 studies including qualitative data on implementation factors, demonstrating as the current literature has suggested, that there is an evidence gap of practice-based implementation studies in community settings. In addition, this may be indicative of publication bias. Given the unique insight and potential value of qualitative research [50, 54, 55], this review indicates that more research employing mixed methods or solely qualitative methods is needed to expand on the literature base for this specific area of implementation science.

### Limitations

This review has some limitations. The search strategy used did not include any methods for identifying grey literature or studies that were published in languages other than English. By not including these as sources, valuable information may have been overlooked. Furthermore, due to the focus of this review, implementation factors, i.e. barriers and facilitators, articles were not selected based on the quality of their effectiveness, and some articles did not report on effectiveness at all. However, the included studies were all published in peer-reviewed journals, indicating that they underwent an academically rigorous review and publication process. The review also excluded studies that focused specifically on populations within the community who have a disability or mental health condition. This may have omitted findings that would be useful for those implementing interventions in these populations and further research is recommended. This review synthesises studies of community-based physical activity interventions, which includes populations of all ages. As such, the barriers and facilitators presented in this review may not apply across all age ranges, with more specific implementation factors relevant to specific age populations such as adolescents or older adults. Finally, this review presents secondary data from evaluations and analyses of interventions, as the necessary qualitative data on implementation factors was only available from studies who had undergone this intervention implementation review process.

## Conclusion

This review identified many facilitators and barriers of implementing physical activity interventions in the community, as well as recommendations for implementation. The findings of this review add to the limited practice evidence base and indicate a similarity in the barriers and facilitators of implementing PA interventions across various contexts/settings including those based in the community. This review suggests that barriers to implementation could have been negated or reduced by an implementation plan in which several evidence-based strategies are embedded. Although several frameworks and guides exist, there is a lack of evidence of the effectiveness of current implementation strategies. Further research is still needed to identify effective implementation strategies in practice settings and provide evidence for successfully engaging key stakeholders at an individual level as part of these strategies. The impact of individuals on the successful implementation of an intervention is undervalued and as such, more consideration is needed for the influence individuals and stakeholders at different levels have on program implementation, particularly where individuals value the intervention. This review also identified recommendations from the included studies, which can be used pragmatically moving forward to guide research and practice in this field, with a particular focus on the processes of implementation.

## Supplementary Information

**Additional file 1.** Search Strategy. This file includes the search syntax and a sample search on SCOPUS.

**Additional file 2.** Data extraction table & Risk of bias. This table presents the data extracted from the studies included in this review (characteristics, facilitators, barriers and recommendations extracted into CFIR domains) and a risk of bias assessment using the Mixed Methods Assessment Tool (MMAT) (Hong et al., 2018).

**Additional file 3.** Barriers and facilitators to implementation. A table with the barriers and facilitators to implementing community-based physical activity interventions presented under the 5 domains of the CFIR and with supporting data from the included studies.

**Additional file 4.** Recommendations for implementation. A table with recommendations for implementing community-based physical activity interventions extracted from the included studies and presented under the 5 domains of the CFIR.

**Additional file 5.** PRISMA 2020 Checklist. A checklist comprising of the 27-items to be included in a systematic review according to the PRISMA 2020 statement.

## Data Availability

All data analysed during this study are included in this published article [and its supplementary information files].

## Abbreviations

PA: Physical Activity
CFIR: Consolidated Framework for Implementation Research
ISPAH: International Society for Physical Activity and Health
I-PARC: Irish Physical Activity Collaboration
WHO: World Health Organisation
PRISMA: Preferred Reporting Items for Systematic Reviews and Meta-Analyses
