# Supplementary material for: Barriers and facilitators to implementing community-based physical activity interventions: a qualitative systematic review
Source: Int J Behav Nutr Phys Act. 2021 Sep 7;18:118. doi: 10.1186/s12966-021-01177-w (PMC8422651; doi:10.1186/s12966-021-01177-w)
Supplement: Supplementary file 1 — Additional file 1. Search Strategy. This file includes the search syntax and a sample search on SCOPUS. [file 12966_2021_1177_MOESM1_ESM.docx]

***Additional file 1 – Search Strategy***

|  | **Syntax Combinations** |
| --- | --- |
| 1 | (implement* OR execut* OR perform* OR deliver* OR process* OR disseminat*) |
| 2 | (“community based” OR community OR population OR society OR neighborhood OR communal OR municipal) |
| 3 | 1 OR 2 |
| 4 | (“physical activity” OR “physical (fit* or train* or active* or endur*))” OR exercis* OR sport* OR “physical education” OR walk* OR cycl* OR activit* OR “public health” OR “health promotion”) |
| 5 | (interve* OR strategy* OR plan OR program* OR polic* OR approach* OR scheme OR framework OR guideline OR concept) |
| 6 | 4 AND 5 |
| 6 | (“process evaluation” OR process* OR plan* OR engag* OR collab* OR eval* OR monitor*) |
| 7 | (outcom* OR fidelity OR feasib* OR adapt* OR sustain* OR “scale up” OR scalability OR scaling OR adopt* OR uptake) |
| 8 | 6 OR 7 |
| 9 | (factor OR aspect OR characteristic OR determinant) |
| 10 | (barrier OR limitat* OR imped* OR restrict* OR difficult*) |
| 11 | (facilitat* OR enable* OR ease OR assist*) |
| 12 | 9 AND (10 OR 11) |
| 13 | 3 AND 6 AND 8 AND 12 |
| 14 | NOT ("active trav*" OR "active trans*" OR "active comm*") |
| 15 | NOT (rehab* OR recovery OR therap* OR care OR diet) |
| 16 | 13 NOT (14 AND 15) |
| 17 | Limit 16 to 2000-2020 AND English |

Sample search (from SCOPUS)

( TITLE ( ( "physical activity" OR "physical* (fit* or train* or active*))" OR exercis* OR sport* OR "physical education" OR walk* OR cycl* ) AND ( interve* OR strategy* OR plan OR program* OR polic* OR approach* OR scheme OR framework OR guideline ) ) ) AND ( TITLE-ABS ( implement* OR execut* OR perform* OR deliver* OR process ) ) AND ( TITLE-ABS ( "community based" OR community OR population OR society OR neighbourhood OR communal OR municipal ) ) AND ( TITLE-ABS ( ( "process evaluation" OR process* OR plan* OR engag* OR collab* OR eval* OR monitor* ) OR ( outcom* OR fidelity OR feasib* OR adapt* OR sustain* OR "scale up" OR scalability OR scaling OR adopt* OR uptake OR utilis* OR utiliz* ) ) ) AND ( TITLE-ABS ( ( factor OR aspect OR characteristic OR determinant ) AND ( ( barrier OR limitat* OR imped* OR restrict* OR difficult* ) OR ( facilitat* OR enable* OR ease OR assist* ) ) ) ) AND NOT ( TITLE-ABS-KEY ( "active trav*" OR "active trans*" OR "active comm*" ) AND TITLE-ABS-KEY ( rehab* OR recovery OR therap* OR care OR diet* ) ) AND PUBYEAR > 1999
