# Supplementary material for: Barriers and facilitators to implementing community-based physical activity interventions: a qualitative systematic review
Source: Int J Behav Nutr Phys Act. 2021 Sep 7;18:118. doi: 10.1186/s12966-021-01177-w (PMC8422651; doi:10.1186/s12966-021-01177-w)
Supplement: Supplementary file 3 — Additional file 3. Barriers and facilitators to implementation. A table with the barriers and facilitators to implementing community-based physical activity interventions presented under the 5 domains of the CFIR and with supporting data from the included studies. [file 12966_2021_1177_MOESM3_ESM.docx]

***Additional file 3: Barriers & Facilitators to Implementation***

| 1. **Intervention Characteristics** | |
| --- | --- |
| **Barriers** | **Facilitators** |
| B1.1 Name of intervention   - The name was unclear and caused reluctance to participate in the programme at district level^3^   B1.2 Lack of evidence base   - the strong advocacy and regional policy framework did not counter-balance the lack of evidence base in the programme^2^ - Lack of evidence-based treatment programs^4^   B1.3 Adaptability – conflict between standardisation vs tailoring to context   - Discrepancy between recommendation to standardise and simplify the intervention and the need to tailor the strategies to local environmental, social and cultural aspects^4^ - Tools and demands not being adaptable to context - doesn't fit well or unrealistic^9^ - Programme content: more focus needed on pragmatic issues, more focus on needs of different schools at start of programme^3^ - A single programme cannot be transferred to all contexts^3^ - Adaptation: Variety of implementation practices in a non-trial setting, which result from various spatial (e.g. playground area) and temporal (e.g. time of year, time of day and/or weather) constraints as well as the presence or absence of other physical activities in the school. The intervention required extensive input from public health department to address^8^   B1.4 Safety Consideration   - Unable to ensure safety and security of participants^6^   B1.5 Physical and temporal barriers   - Spatial and temporal constraints acted as key barriers that prevented the intervention from taking place^8^ - Physical barriers such as several flights of stairs or restricted outdoor space^8^   B1.6 Failures in new technology implemented   - Failures in new information and communication technology support tools integrated and delays in fixing them has a negative effect^9^ | F1.1 Cost to participant   - Free or affordable^1^ - Low cost^5^ - affordable for target participants^6^   F1.2 Cost to organisation   - No special equipment needed^5^ - requires little start-up equipment^6^ - Secured infrastructure (church halls and gymnasiums) that can be used at no or low cost^1^   F1.3 Pragmatic and clear programme content   - Programme content had a positive effect on appreciation due to pragmatic and concrete nature^3^ - clear protocols, task descriptions^4^ - well-defined program components^4^   F1.4 Adaptability (both by implementers and to context)   - potential for tailoring^4^ - More room for initiative on the part of district teams led to higher implication of trainers/training^3^ - Context-specific health activities^1^ - Activity coaches were able to make small changes to program to facilitate implementation (such as content due to time constraints/relevance to context)^11^ - Program content allowed for modification where necessary: improvised using other equipment where it was lacking for certain exercises^6^ - Adaptability of recruitment strategies to target population and context^10^ - adapting to community context^11^ - opportunity for local tailoring^12^ - program components adaptable to participants' abilities and age levels^6^   F1.5 Programme compatibility with staff and participants   - Programme was considered timely, necessary, relevant and scalable^11^ - length of program isn't overwhelming^5^ - Offer unique program within communities in which it was implemented^6^ - Geographically accessible^6^ - Compatibility of intervention tasks with PE teachers and city district sports coordinators regular function^4^ - No special clothing or equipment needed^8^ - Easily fits within participant's lifestyles^5^   F1.6 Development and availability of innovative information and communication technologies^7^  F1.7 Credibility from evidence source   - Evidence strength and quality strongly distinguished between high and low implementation performances^9^ - Evidence source was a factor contributing to sustainability of program (scientific support and credibility)^6^ - Evidence-base developed by researchers made CTM more credible for those delivering programme and for participants^11^   F1.8 Positive perception of intervention implementer by participants   - role of the provider in delivering the intervention was one that was perceived positively by participants^14^   F1.9 Sustainability of the intervention   - The concepts of success and failure centre around the issue of sustainability^6^ - Critical areas for optimisation to enhance the feasibility and sustainability^7^ |

| 1. **Inner Setting** | |
| --- | --- |
| **Barriers** | **Facilitators** |
| B2.1 Competing priorities   - Competing priorities for leadership: difficult to initiate and maintain a leadership role in health promoting activities^1^ - Identification of other issues as priority, shifting the focus from health education and physical activity^2^ - conflicting aims with members who don't identify with the same agenda^10^ - Competing demands/expectations of organisations was a major barrier to implementation^8^   B2.2 High staff turnover   - no new team to replace outgoing^2^ - loss of information^2^ - High staff turnover^2^ - Personnel turnover^4^ - In smaller communities it was difficult to find and retrain fitness leaders^11^   B2.3 Lack of communication within the team   - lack of coordination between staff^12^ - Lack of communication within the team^2^   B2.4 Lack of support from leadership^2^  B2.5 Lack of funding   - Lack of funding - Leaders indicated that their organisations have the infrastructure and capacity but not the financial resources^11^   B2.6 Implementing intervention from obligation   - Where a policy is new, organisations may implement more from institutional obligation rather than coordinated action that makes sense in their organisation.^2^ - not having an implementation team in place, so that people participated out of duty but without the intention to continue the project^2^   B2.7 Staff burnout   - Organisational climate: staff who experience full professional burn- out or degrees of professional exhaustion would not devote any extra time for projects or actions^3^ - Staff feel pressurised by work and time constraints^6^ - staff not feeling their involvement in program is worthwhile affects sustainability of intervention^6^   B2.8 Lack of perceived responsibility and motivation among organisations^4^  B2.9 Limited capacity to take part in multiple initiatives   - management view: organisations have limited capacity to take part in multiple initiatives. How the intervention fits into existing timetables that are already full^8^ - over-solicitation of organisations to participate in research lead to non-response to research team^2^ - All the schools from same school district could not be enrolled which led to tensions and reluctance^3^   B2.10 High level of organisation needed   - High level of organisation needed can lead to confusion when this is lacking and losing sight of program goal^9^ - underestimation of the efforts needed to attract participants led to poor attendance at informational meetings^4^ - lack of effective delivery of training to staff which might help to facilitate sustainable walking programmes for targeted groups^10^ - Lack of time for preparation^12^   B2.11 Too much change required to implement   - Some activities required too much of a change compared to previous practices in the area - difficulties integrating into normal practice^12^   B2.12 Poor staff training quality   - Awkward language used in training materials for staff and heavy workload^7^ | F2.1 Support and commitment from leadership   - In organisations where existing projects were extended, support from the project leader promoted sustainability^2^ - Leadership and modelling: leadership to take lead role in promoting physical health and be role models^1^ - Commitment from leadership to provide health education to participants^1^ - Support from the project leader promoted sustainability^2^ - buy-in from leaders was essential factor^6^   F2.2 Clear information and communication strategies within organisations   - Clear information and communication strategies within organisations^4^ - organisational environment of intervention provider as contributing to its sustainability^6^   F2.3 Provider training and capacity building   - Each provider received training to facilitate effective delivery of the intervention^14^ - Competence development program^12^ - Training facilitated coordination^3^   F2.4 Strong shared commitment and sense of ownership   - strong commitment and motivation from organisation to comply with shared goal^4^ - Sense of ownership from staff is fundamental to successful implementation^2^ - Existing culture of working together helped to facilitate implementation^6^ - Sense of belonging within each branch^6^ - In organisations where new projects were implemented, group participation created common ground, and a shared understanding and vision of the health promoting role of organisation^2^   F2.5 Feedback to staff   - Providing monthly feedback led to staff valuing it and being encouraged to reflect and prompted team discussions to identify problems and look for solutions^9^   F2.6 Easy to integrate intervention goals within existing structures   - easy to integrate intervention goals, methods, procedures and tasks within organisation^4^ - Connections with existing structures such as coffee meetings increased attendance at intervention informational meetings for participants^4^ - Having coaches from within the organisation helped with implementation of the program - coordinators already have a relationship with them^11^ - Leaders noted complete alignment with their organisational priorities, vision and strategic directions^11^   F2.7 Strong staff relationships   - Development of relationships between staff counterbalanced fact that programme was imposed by staff at higher decision-making levels (management)^3^ |

| 1. **Outer Setting** | |
| --- | --- |
| **Barriers** | **Facilitators** |
| B3.1 Cultural barriers   - cultural barriers within communities^6^ - Conflict between church and state: leaders are concerned about maintaining separation between the two and may choose to not participate in health programs funded by the government^1^   B3.2 Instability or lack of policies supporting target group   - Instability in the policies and institutional environments in the school setting^2^ - Lack of policy regarding target group^4^   B3.3 Poor relationship between organisation and community   - Fact that programme was initiated by a community-made decision did not strengthen the weak ties between the implementing organisation and the community.^3^ - quality of participant-organisational relationship seemed to overpower the beneficial effect of leadership on the part of the school head where the relationships between participants and staff were very poor^3^ - Cooperation among all the Public Health Centre staff and linkage with community agents are extremely challenging and complex social processes^7^   B3.4 Lack of community buy-in   - Communicating with participants the importance of programme outcomes was a challenge^6^ - Challenges with participant acceptance of program^12^ - Lack of community buy-in^6^   B3.5 Lack of coordination and communication between organisations   - Complexity of programme requiring multidisciplinary collaboration between organisations^4^ - lack of overall coordination hampered communication between organisations^4^   B3.6 Funding between collaborating organisations   - Limited resources and funding from funding partners to support effective recruitment^10^ - Complex financial structures between collaborating organisations^4^   B3.7 Availability of resources   - poor availability and accessibility of necessary facilities^4^ - finding suitable and affordable venues was a difficulty^6^ - resource availability^10^ - Lack of resources^6^ - Lack of existing sports clubs^4^ | F3.1 Participation of stakeholders in decision-making process   - active participation of appropriate stakeholders in the decision-making process to adapt the intervention to a specific context^7^ - tapping into the cultural norms and behaviours of potential participants in target context^10^   F3.2 Funding   - Availability of financial resources^4^ - Government funding to help improve access to health resources and enhance delivery of health programs^1^   F3.3 Accessible to communities in which it was implemented^6^  F3.4 Community involvement to support the intervention   - Community involvement as a reinforcing factor to facilitate program^1^ - Local support^2^ - linkage with community resources^7^ - Support from local authority^8^ - Motivation and commitment of community members helped further facilitate implementation^6^   F3.5 High perceived fit of intervention in policy goals/agendas   - Policy formalising health education providing obligation of schools to implement school projects which include health and well-being objectives in addition to educational objectives^2^ - High perceived fit of intervention in policies^4^   F3.6 Political advocacy and support   - School Health Promotion Committees created by Ministry of Education as institutional supporting tool to empower local schools and initiate collaborations between policy makers and practitioners and researchers^2^ - Institutional support from head of regional education authority promoted active participation^3^ - Linkages with Ministry of Health as essential for scale-up and possible mechanism to ensure sustainability^11^ - Political advocacy and support^4^   F3.7 Effective communication strategies between stakeholders   - Effective cooperation between school, city district and sports clubs^4^ - formalisation of partnerships^4^ - continued participation of all partners^4^ - Created a sense of unity through working towards a common goal^6^ - Trust and motivation which partner organisations build up with their clients^10^ - The relationship between school staff and district level decision-makers of school management influenced the opportunities for school staff to show ownership and develop action^3^ - Effective communication strategies between stakeholders^4^   F3.8 Volunteerism   - Spirit of volunteerism^1^   F3.9 Role of support and research system   - Role of support and research system was viewed as instrumental to successful implementation^11^   F3.10 Leadership and buy-in from range of stakeholders   - Strong leadership and active engagement by a range of stakeholders facilitated implementation^11^ - Business and newspapers are huge supporters^5^ |

| 1. **Individual Characteristics** | |
| --- | --- |
| **Barriers** | **Facilitators** |
| B4.1 Lack of motivation   - lack of motivation in colleagues^2^ - motivation^3^   B4.2 Lack of knowledge   - need for more info and clarity about tasks among staff - Lack of knowledge of training or intervention^2^   B4.3 Perceived imposed participation in intervention training   - negative effect: Participation was sometimes seen as being imposed^3^   B4.4 Values inconsistent with lifestyle/context   - values conveyed by the programme were inconsistent with the reality of school life^3^   B4.5 Lack of perceived importance of communicating with participants by organisation staff^4^  B4.6 Perceived workload among staff^4^  B4.7 Poor participant attitude^4^  B4.8 Challenge to find committed leaders   - Challenging to find committed intervention leaders who are able to lead without organisation staff is a challenge^6^ | F4.1 Well-trained   - Inclusion of training in programme had positive effect as staff felt programme was in line with their expectations^3^ - Agents understood the basic functioning principles of the programme^5^ - realistic expectations about tasks and responsibilities^4^ - workshops were significant for individual motivation^12^ - Well trained^6^   F4.2 Dedicated   - Personal effort^9^ - Dedicated^6^   F4.3 Leaders take ownership of problem addressed by intervention   - Pivotal role of activity coach in delivery of intervention - augmented when coaches took ownership of programme^11^ - Feeling of ownership of programme for participants^9^ - Leaders take ownership of problem^1^   F4.4 Leaders motivate others   - Leaders providing a new impulse and preventing the team from losing motivation, and drop out^2^   F4.5 High individual motivation   - Intense motivation of the city district's sports coordinator and PE teachers^4^ - Motivated^6^   F4.6 High perceived importance of intervention by staff   - Perception of members, intervention leaders and organisational staff that intervention uplifts communities through their involvement^6^ - Perceived value in the approach of the implementation of the intervention even though it differed from usual practice^14^ - high perceived importance of intervention goals and participation^4^   F4.7 Positive attitudes and beliefs   - Positive attitudes and encouragement from coaches make big difference to participants^11^ - Passionate^6^ - positive attitude and beliefs of political stakeholders^4^ - positive attitude toward adaptations and changes in policies and practices^4^   F4.8 Members and leaders experienced increases in self-efficacy^6^  F4.9 Feeling empowered^6^  F4.10 Strong feeling of reward from engaging across all levels   - Strong feeling of reward from participating as a coach^11^ - Wanting to be involved^6^ - Staff and management could identify a wide range of benefits from engaging with the scheme^8^ - Leaders have knowledge of benefits of engaging^1^ |

| 1. **Processes of Implementation** | |
| --- | --- |
| **Barriers** | **Facilitators** |
| B5.1 Insufficient means allocated for implementation   - Recreation coordinators underestimated the time and effort to recruit and deliver in many communities^11^ - Lack of time to coordinate organisational procedures in order to increase efficiency^4^ - Insufficient means were allocated which hindered the implementation of the training^3^   B5.2 Complexity of intervention   - Statistical demands of program - doesn’t reflect reality and is very stressful^9^ - Complexity of intervention causing delay in preparation period^4^   B5.3 Top-down implementation strategy   - Top-down implementation/initiation mode impacted implementation greatly in a negative way^3^ - Institutional support from head of regional education authority meant implementation was perceived as following top-down mode and adding constraints^3^ | F5.1 Engaging key stakeholders in decision-making throughout whole implementation process (including pre-delivery of intervention)   - Engagement of whole organisation team and community members involved in shared decision-making and cooperation in a community-based program^7^ - Empowering communities through consultation process where their needs were identified^6^ - engaging appropriate individuals in the implementation and use of the intervention (Engaging with stakeholders outside of the intervention setting)^1^ - Bottom-up process of involving key stakeholders in discussion, consensus and a decision-making process about priorities according to specific context and about the workflow and role and contributions of different members can lead to a greater sense of ownership and commitment to adhering to the program^9^ - Co-designed with community partners over 2 years^11^   F5.2 Appointed community-based members as leaders   - Appointed community-based members as leaders was viewed as empowering and important for maintaining continuity^6^   F5.2 Designed using existing resources and context characteristics   - Design which takes into account the specific characteristics of the context - enables the selection of the appropriate approach for a given school, which makes the best of existing resources, creates a team and generally focusses on what will make a difference^2^ - Existing community infrastructure was seen to facilitate the intervention implementation process^6^   F5.3 Involvement of experts to tailor intervention   - Involvement of experts in tailoring the program to the school characteristics^4^ - Support from experts^4^   F5.4 Using theoretical model to inform recruitment strategies   - Having a theoretical model to inform recruitment process and tailor strategies to target groups^10^   F5.6 Support and research lead checking in with program staff facilitated problem solving and feedback loops^11^  F5.7 Use of wide variety of strategies to implement the intervention^11^  F5.8 Enough time for preparation before delivery   - Ensure adequate time to promote programme, generate materials and conduct training^11^   F5.9 Collaborative effort built into design   - support from intervention team^4^ - Collaborative effort^9^ - Social support is the most useful aspect of programme^1^ - Accountability between participants^5^ |

**References:**

1. Banerjee, A. T., Kin, R., Strachan, P. H., Boyle, M. H., Anand, S. S., & Oremus, M. (2015). Factors Facilitating the Implementation of Church-Based Heart Health Promotion Programs for Older Adults: A Qualitative Study Guided by the Precede-Proceed Model. *American journal of health promotion*, *29*(6), 365–373. <https://doi.org/10.4278/ajhp.130820-QUAL-438>
2. Darlington, E.J., Simar, C., & Jourdan, D. (2017). Implementation of a health promotion programme: a ten-year retrospective study. *Health Education*, *117*(3), 252-279. doi:10.1108/HE-09-2016-0038
3. Darlington, E.J., Violon, N., & Jourdan, D. (2018). Implementation of health promotion programmes in schools: an approach to understand the influence of contextual factors on the process? *BMC Public Health*, *18*(163). DOI:10.1186/s12889-017-5011-3
4. de Meij, J. S., van der Wal, M. F., van Mechelen, W., & Chinapaw, M. J. (2013). A mixed methods process evaluation of the implementation of JUMP-in, a multilevel school-based intervention aimed at physical activity promotion. *Health promotion practice*, *14*(5), 777–790. doi:10.1177/1524839912465750
5. Downey, S. M., Wages, J., Jackson, S. F., & Estabrooks, P. A. (2012). Adoption decisions and implementation of a community-based physical activity program: a mixed methods study. *Health promotion practice*, *13*(2), 175–182. <https://doi.org/10.1177/1524839910380155>
6. Draper, C. E., Kolbe-Alexander, T. L., & Lambert, E. V. (2009). A retrospective evaluation of a community-based physical activity health promotion program. *Journal of physical activity & health*, *6*(5), 578–588. <https://doi.org/10.1123/jpah.6.5.578>
7. Grandes, G., Sanchez, A., Cortada, J. M., Pombo, H., Martinez, C., Balagué, L., Corrales, M. H., de la Peña, E., Mugica, J., Gorostiza, E., & PVS group (2017). Collaborative modeling of an implementation strategy: a case study to integrate health promotion in primary and community care. *BMC research notes*, *10*(1), 699. <https://doi.org/10.1186/s13104-017-3040-8>
8. Hanckel, B., Ruta, D., Scott, G., Peacock, J.L., & Green, J. (2019). The Daily Mile as a public health intervention: a rapid ethnographic assessment of uptake and implementation in South London, UK. *BMC Public Health,* *19*(1167). <https://doi.org/10.1186/s12889-019-7511-9>
9. Martinez, C., Bacigalupe, G., Cortada, J. M., Grandes, G., Sanchez, A., Pombo, H., Bully, P., & PVS group (2017). The implementation of health promotion in primary and community care: a qualitative analysis of the 'Prescribe Vida Saludable' strategy. *BMC family practice*, *18*(1), 23. <https://doi.org/10.1186/s12875-017-0584-6>
10. Matthews, A., Brennan, G., Kelly, P., McAdam, C., Mutrie, N. & Foster, C. (2012). A qualitative study of recruitment approaches in community based walking programmes in the UK. *BMC Public Health,* *12*(635). <https://doi.org/10.1186/1471-2458-12-635>
11. Sims-Gould, J., McKay, H.A., Hoy, C.L., Nettlefold, L., Gray, S.M., Lau, E.Y., & Bauman, A. (2019). Factors that influence implementation at scale of a community-based health promotion intervention for older adults. *BMC Public Health,* *19*(1619). https://doi.org/10.1186/s12889-019-7984-6
12. Smedegaard, S., Brondeel, R., Christiansen, L.B., & Skovgaard, T. (2017). What happened in the ‘Move for Well-being in School’: a process evaluation of a cluster randomized physical activity intervention using the RE-AIM framework. *Int J Behav Nutr Phys Act,* *14*(159). <https://doi.org/10.1186/s12966-017-0614-8>
13. Williams, S. L., McSharry, J., Taylor, C., Dale, J., Michie, S., & French, D. P. (2020). Translating a walking intervention for health professional delivery within primary care: A mixed-methods treatment fidelity assessment. *British journal of health psychology*, *25*(1), 17–38. https://doi.org/10.1111/bjhp.12392
