# Supplementary material for: Barriers and facilitators to implementing community-based physical activity interventions: a qualitative systematic review
Source: Int J Behav Nutr Phys Act. 2021 Sep 7;18:118. doi: 10.1186/s12966-021-01177-w (PMC8422651; doi:10.1186/s12966-021-01177-w)
Supplement: Supplementary file 4 — Additional file 4. Recommendations for implementation. A table with recommendations for implementing community-based physical activity interventions extracted from the included studies and presented under the 5 domains of the CFIR. [file 12966_2021_1177_MOESM4_ESM.docx]

***Additional file 4: Recommendations for Implementation***

| 1. **Intervention Characteristics** |
| --- |
| R1.1 Using policy as a formidable tool for health promotion   - Policy can be a formidable tool for health promotion as long as its understanding and development is consistent with implementation in the specific context.^1^   R1.2 Potential barriers need to be anticipated and addressed before implementation^2^   - Suggest an extensive assessment of the specificities of the intervention context and its surrounding environment is required prior to any form of programme implementation   R1.3 Programme components – standardised, simple, scalable and renewed   - standardised, simplified and scalable program components^3^ - reduction of complexity in content and delivery^3^ - Program materials need to be kept fresh for agents so program doesn't seem repetitive^4^   R1.4 Maintain cost to participant (do not increase)^4^  R1.5 Plan for scale up^5^  R1.6 Innovative ways of motivating participants not ready to change and ensuring continuity of care for those with intention to change behaviour to minimize false expectations^5^  R1.7 Using ongoing research to assist organisations in maintaining fidelity to core principles^6^  R1.8 Assess fidelity of delivery   - Recording sessions to assess fidelity of delivery can in itself serve to enhance fidelity of delivery^9^ |

| 1. **Inner Setting** |
| --- |
| R2.1 Improvement of efficiency and reliability of the information and communication tools and databases   - Coordinated working is needed at all professional levels, to foster communication between different tiers of professionals and to provide sufficient staff resources^5^ - improvement of efficiency and reliability of the information and communication tools and databases^5^ |

| 1. **Outer Setting** |
| --- |
| R3.1 Need for local support   - Need for local support and consultancy services for implementation^1^   R3.2 Need for policy development   - Need for policy development is apparent^1^ - Include in state legislation^4^   R3.3 Needs long-term funding   - Needs to provide long-term funding and provisions of trained workforce who can support local level policy development and implementation^1^   R3.4 Formalised coalition   - formalised coalitions, integration of policy and synchronisation of tasks and protocols^3^   R3.5 Increase media support^4^  R3.6 Easier communication to participants^4^  R3.7 Improve coordination to avoid duplication   - at community level: improvement of coordination with community resources to align forces and avoid duplication of efforts^5^   R3.8 Need to work in partnership with organisations and agencies working in target groups, especially in hard to recruit groups^7^  R3.9 More research focus is needed on fidelity to implementation strategies^8^ |

| 1. **Individual Characteristics** |
| --- |
| R4.1 More attention for stakeholders’ skills and involvement across contexts is recommended to improve self-efficacy^3^ |

| 1. **Processes of Implementation** |
| --- |
| R5.1 Planning with clear steps for implementation   - smart planning and control by clear communication and feedback instruments^3^ - need to give attention to programme initiation modes and emphasize importance of negotiated planning^2^ - stepwise implementation^3^   R5.2 Collaboration between all aspects of community and setting from start of programme implementation and before programme is introduced. This introduces complexity to the process^2^  R5.3 Use of social marketing principles^3^  R5.4 Maintain program champion^4^  R5.5 Maintain ease of delivery^4^  R5.6 Understanding if and how these decisions are made and what trade-offs are made at the different levels of the intervention is important for understanding intervention implementation^6^  R5.7 Intensity of contact between research team and providers may have contributed to level of adherence^9^ |

**References**

1. Darlington, E.J., Simar, C., & Jourdan, D. (2017). Implementation of a health promotion programme: a ten-year retrospective study. *Health Education*, *117*(3), 252-279. doi:10.1108/HE-09-2016-0038
2. Darlington, E.J., Violon, N., & Jourdan, D. (2018). Implementation of health promotion programmes in schools: an approach to understand the influence of contextual factors on the process? *BMC Public Health*, *18*(163). DOI:10.1186/s12889-017-5011-3
3. de Meij, J. S., van der Wal, M. F., van Mechelen, W., & Chinapaw, M. J. (2013). A mixed methods process evaluation of the implementation of JUMP-in, a multilevel school-based intervention aimed at physical activity promotion. *Health promotion practice*, *14*(5), 777–790. doi:10.1177/1524839912465750
4. Downey, S. M., Wages, J., Jackson, S. F., & Estabrooks, P. A. (2012). Adoption decisions and implementation of a community-based physical activity program: a mixed methods study. *Health promotion practice*, *13*(2), 175–182. <https://doi.org/10.1177/1524839910380155>
5. Grandes, G., Sanchez, A., Cortada, J. M., Pombo, H., Martinez, C., Balagué, L., Corrales, M. H., de la Peña, E., Mugica, J., Gorostiza, E., & PVS group (2017). Collaborative modeling of an implementation strategy: a case study to integrate health promotion in primary and community care. *BMC research notes*, *10*(1), 699. <https://doi.org/10.1186/s13104-017-3040-8>
6. Hanckel, B., Ruta, D., Scott, G., Peacock, J.L., & Green, J. (2019). The Daily Mile as a public health intervention: a rapid ethnographic assessment of uptake and implementation in South London, UK. *BMC Public Health,* *19*(1167). <https://doi.org/10.1186/s12889-019-7511-9>
7. Matthews, A., Brennan, G., Kelly, P., McAdam, C., Mutrie, N. & Foster, C. (2012). A qualitative study of recruitment approaches in community based walking programmes in the UK. *BMC Public Health,* *12*(635). <https://doi.org/10.1186/1471-2458-12-635>
8. Sims-Gould, J., McKay, H.A., Hoy, C.L., Nettlefold, L., Gray, S.M., Lau, E.Y., & Bauman, A. (2019). Factors that influence implementation at scale of a community-based health promotion intervention for older adults. *BMC Public Health,* *19*(1619). https://doi.org/10.1186/s12889-019-7984-6
9. Williams, S. L., McSharry, J., Taylor, C., Dale, J., Michie, S., & French, D. P. (2020). Translating a walking intervention for health professional delivery within primary care: A mixed-methods treatment fidelity assessment. *British journal of health psychology*, *25*(1), 17–38. https://doi.org/10.1111/bjhp.12392
